# Electrospinning of Metal–Organic Frameworks for Energy and Environmental Applications

**DOI:** 10.1002/advs.201902590

**Published:** 2019-12-11

**Authors:** Yibo Dou, Wenjing Zhang, Andreas Kaiser

**Affiliations:** ^1^ Department of Energy Conversion and Storage Technical University of Denmark Anker Engelunds Vej, Building 301 DK‐2800 Kongens Lyngby Denmark; ^2^ Department of Environmental Engineering Technical University of Denmark Miljøvej 113 DK‐2800 Kongens Lyngby Denmark

**Keywords:** electrospinning, energy and environmental applications, hierarchical porous structure, metal–organic frameworks

## Abstract

Herein, recent developments of metal–organic frameworks (MOFs) structured into nanofibers by electrospinning are summarized, including the fabrication, post‐treatment via pyrolysis, properties, and use of the resulting MOF nanofiber architectures. The fabrication and post‐treatment of the MOF nanofiber architectures are described systematically by two routes: i) the direct electrospinning of MOF‐polymer nanofiber composites, and ii) the surface decoration of nanofiber structures with MOFs. The unique properties and performance of the different types of MOF nanofibers and their derivatives are explained in respect to their use in energy and environmental applications, including air filtration, water treatment, gas storage and separation, electrochemical energy conversion and storage, and heterogeneous catalysis. Finally, challenges with the fabrication of MOF nanofibers, limitations for their use, and trends for future developments are presented.

## Graphical abstract

Metal–organic framework based nanofibers with hierarchical porous structure fabricated via electrospinning are reviewed for energy and environmental applications.

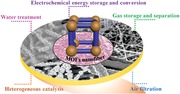

## 1 Introduction

Worldwide growth of population and energy consumption is causing a significant increase in CO_2_ and other greenhouse gas emissions, resulting in climate change and the pollution of the environment.^1^, ^2^, ^3^, ^4^ A variety of emerging technologies, such as devices for energy conversion and storage, or for advanced air and water cleaning, are developed to meet the large energy demands and tackle various environmental challenges.^5^, ^6^, ^7^ Functional porous materials, such as active carbon, porous polymers, zeolites, and metal–organic frameworks (MOFs), play an important role in the improvement of energy and environmental technologies.^8^, ^9^, ^10^, ^11^, ^12^ MOFs are an emerging class of crystalline materials that are composed of metal ions connected by organic linkers with a periodic, nanoscaled structure and ultrahigh surface areas. With their tunable pore/cage sizes, flexible skeleton, and large surface‐to‐volume ratio,^13^, ^14^, ^15^, ^16^, ^17^, ^18^, ^19^ MOFs have a large potential for a wide range of applications, including gas storage and separation, rechargeable batteries, supercapacitors, solar cells, nanoreactors, heterogeneous catalysis, or drug delivery.^20^, ^21^, ^22^, ^23^, ^24^, ^25^, ^26^, ^27^ Recently, significant efforts have been devoted to the design and synthesis of new MOFs structures and the investigation of their physical or chemical properties. MOFs are generally prepared as bulk form through traditional hydrothermal or solvothermal synthesis.^28^, ^29^, ^30^, ^31^ In order to expand the application range, suitable pathways for the structuring of MOF powders into a functional architecture or devices are highly desirable.

Currently, intensive efforts are focusing on the structuring of MOFs at the mesoscopic/macroscopic scale for the use as coatings, membranes, or sophisticated architectures in specific devices and applications.^32^, ^33^, ^34^, ^35^ A major difference in the structuring of MOFs into complex shapes compared to inorganic microporous materials, such as zeolites or silica, is the fact that most inorganic binders cannot be used for MOFs as these binders usually require heat treatment. The intrinsic fragility of MOFs needs to be considered which is related to the inorganic/organic hybrid character of this class of materials and the resulting limited thermal and mechanical stability. Alternatively, a more effective way to structure MOFs is the combination with polymer materials. The polymer which acts as a binder improves the mechanical flexibility and ensures chemical stability. Numerous methods have been developed for structuring MOF‐polymer compositions including hard or soft templates, spin‐ or dip‐coating, spray‐drying, printing, or lithography approaches.^36^, ^37^, ^38^ The integration of MOFs into a polymer matrix can result in poor MOF‐polymer dispersion and compatibility issues owing to the different physical and chemical properties for the two kinds of materials and this is a challenge in some applications, e.g., in MOF‐based mixed matrix membranes for gas separation.^39^, ^40^, ^41^, ^42^ The poor interfacial compatibility would lead to agglomeration of MOF particles, causing the formation of nonselective voids between the MOFs particles and the polymer.

Electrospinning is a fabrication method to produce continuous ultrafine fibers with diameters in the range of a few tens of nanometers to a few micrometers in the form of nonwoven mats, yarns, etc. The mechanism of electrospinning is based on the ejection and elongation of a viscous polymer melt or solution under a high‐voltage electric field which is then solidified in the electrified fluid jet on a collector.^43^, ^44^, ^45^ When the electrostatic forces on the fluid (droplets) overcome the surface tension, the fluid droplet deforms to a conical shape called a Taylor cone and is accelerated to the collector in the form of nanofibers. Factors that influence the electrospinning process and the structure of the resulting nanofibers are the slurry concentration, viscosity, flow rate, voltage, the working distance, humidity, etc. Mostly polymer nanofibers are produced by electrospinning but also metal, ceramic‐based nanofibers have been obtained.^44^, ^45^ Nanofibers have a couple of fascinating properties such as high aspect ratio, large specific surface area to volume, multiscale porosity, and high flexibility. These properties make nanofibers very interesting for a wide range of applications in energy storage and conversion, medical and healthcare, biotechnology to environmental engineering.^46^, ^47^, ^48^, ^49^

Electrospinning has recently been reported as an elegant approach for shaping various MOFs into hybrid materials with multiscale porosity and additional functionalities. In the first pioneering work from 2011 by Ostermann et al.,^50^ the fabrication of MOFs nanofiber by electrospinning a mixture slurry of zeolitic imidazolate framework‐8 (ZIF‐8) and polyvinylpyrrolidone (PVP) was reported. MOF/polymer nanofibers with hierarchical porous structure combine the advantages of both types of materials, including structural flexibility, light weight, large surface area‐to‐volume ratio, high porosity, and tunable pore size at varied length scale.^51^, ^52^, ^53^, ^54^, ^55^ The highly open and interconnected nanofiber structure allows excellent access of fluids, e.g., low transport limitations for gases and liquids. In addition, the polymer matrix allows the shaping, improves the handling, deployment, and regeneration of the composite materials. This unique combination of properties makes porous MOF nanofiber structures highly interesting in the fields of energy and environmental applications.

**Figure 1:**
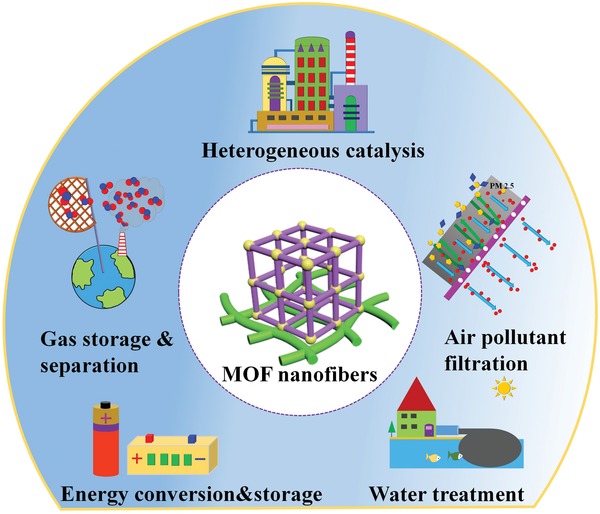
Schematic illustration of the potential use of MOF nanofibers in various energy and environmental applications.

The synthesis, chemical modification, and potential applications of MOFs have been widely reviewed previously.^56^, ^57^, ^58^, ^59^ A complete overview of the structuring of functional MOFs into hierarchical porous nanofibers by electrospinning for energy and environment applications has not yet been reported despite an increasing relevance of this class of materials. However, important work on MOF‐derived carbon nanofibers (CNFs) for use in energy applications has been recently reviewed by the group of Yamauchi.^60^ Given the rapid progress in this field, this review aims to fill this gap and provide a complete overview and update on the development of MOF nanofiber materials in energy and environmental applications (**Figure** **1**). The following discussion is intended to give the reader a detailed coverage on the fabrication, the properties, and the applications of MOF nanofiber structures. The fabrication of MOF nanofibers and their derivatives is conceptually categorized in two routes (Section 2). For each route, the unique functionalities and properties of the obtained MOF nanofiber architectures are discussed. Then, a summary of recently emerging new energy and environmental applications for the MOF nanofibers are reviewed (Section 3). It has to be pointed out that the fabrication and application of derivatives of MOF nanofibers are described in Sections 2.3 and 3.4, which is an important class of novel nanofiber materials, especially for applications in electrochemical energy storage and conversion. Finally, challenges and future opportunities of MOF nanofibers are presented. We hope this review illustrates different strategies to design, fabricate, and use MOF nanofiber structures for specific applications and inspires researchers to tackle future challenges in this area.

## 2 Routes for the Structuring of MOF Nanofiber Architectures

Two major routes have been developed for the structuring of MOF‐polymer nanofiber architectures based on electrospinning, which are “direct electrospinning” and “surface decoration.” MOF nanofibers obtained from both routes can be subsequently converted by post‐treatments into derivatives of MOF nanofibers. We give selected examples for the two routes in Sections 2.1 and 2.2. The fabrication of derivatives of MOF nanofibers is an extra process, which is explained in Section 2.3.

In direct electrospinning, a mixture of a slurry that contains MOF particles and a polymer solution is directly electrospun into a composite nanofiber.^61^, ^62^ The resulting MOFs are embedded in a polymeric nanofiber matrix. However, the inner pores of the MOF particles are often covered by the polymer matrix, which limits the accessibility and activity of the MOF in most applications. The second structuring route is the growth of MOF particles on the surface of the nanofibers.^63^, ^64^ The main advantage of this route is the fact that the surfaces and the inner pores of the pristine MOF crystals are not covered by polymer and are fully accessible. Furthermore, the chemical and physical properties of the MOFs are not altered. The surface decoration route normally requires a two‐step preparation with electrospinning of a polymer nanofiber layer and subsequent growth of the MOF on the nanofiber surfaces and inside the open porosity. This route requires to consider the selection of stable polymer nanofiber structures that can survive the synthesis conditions for those targeted MOF structures, which might require the use of aggressive solvents or relatively high temperatures and pressures (hydrothermal or solvothermal conditions).

### 2.1 Direct Electrospinning of MOF Nanofibers

#### 2.1.1 Fabrication of MOF‐Polymer Nanofibers

**Figure 2:**
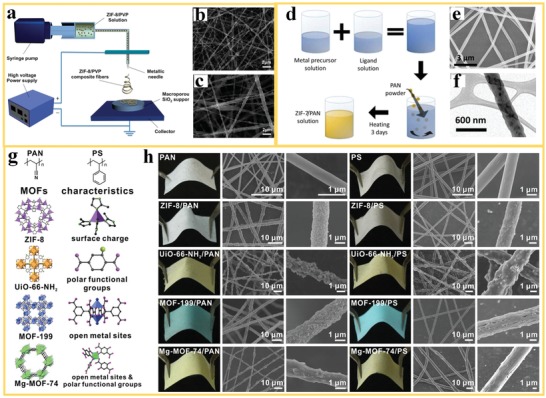
The direct electrospinning of MOF‐polymer composite nanofibers: a) Electrospinning of ZIF‐8‐PVP nanofiber layer on a porous SiO_2_ support (as seeds for further in situ growth of a MOF membrane). b,c) SEM images of the ZIF‐8 crystallites embedded in the PVP nanofibers, with mass fraction of 6 and 12 wt%. Reproduced with permission.^65^ Copyright 2012, Royal Society of Chemistry. d) A schematic for the mixing steps of metal ion and organic ligand precursor solution for electrospinning of freestanding ZIF‐7/PAN nanofibers and e,f) the resulting microstructures by SEM and TEM images. Reproduced with permission.^68^ Copyright 2016, Wiley‐VCH. g) Different types of MOFs with different functionalities, investigated for the use in MOF‐polymer nanofiber layers and h) photos and SEM images of different MOF‐nanofiber composites after electrospinning. Reproduced with permission.^69^ Copyright 2016, American Chemical Society.

MOF‐polymer nanofibers were fabricated initially by a simple three‐step procedure: MOF powder particle synthesis, preparation of a MOF‐polymer slurry, and direct electrospinning of the slurry.^65^, ^66^, ^67^ Depending on the targeted MOF and polymer system for a specific application, the electrospinning of different MOF‐polymer composite materials has been optimized in respect to the choice of the solvent/polymer system, the slurry properties, and the electrospinning conditions. Several polymer‐solvent systems have been chosen for electrospinning, such as polystyrene (PS) in tetrahydrofuran (THF), PVP in ethanol (EtOH), and polyacrylonitrile (PAN) in dimethylformamide (DMF). MOF particles can be mixed with different type of polymer/solvent systems to form a slurry for direct electrospinning as long as chemical compatibility between polymer, solvent, and MOF component is ensured. In this way, different types of MOF‐polymer nanofibers can be produced by optimizing the experimental conditions, such as the concentration of the MOF, the viscosity of the slurry, the voltage, the distance between the needle and the collector. For example, Rose et al.^67^ described this type of process for the fabrication of MOF nanofiber structures with HKUST‐1 (HKUST = Hong Kong University of Science and Technology) and MIL‐100 (Fe) (MIL = Materials of Institut Lavoisier) for use as functional textiles. Composite MOF nanofiber layers can be produced as freestanding structures or deposited on different type of substrates, depending on the application. Fan et al.^65^ utilized in situ growth of ZIF‐8 to coat macroporous SiO_2_ support tubes with MOF nanofiber layers for use in a gas separation membrane. **Figure** **2**a shows schematically the direct electrospinning of a mixture of ZIF‐8 and PVP polymer to produce MOF nanofibers on a SiO_2_ support. Figure 2b,c reveals the microstructure of the MOF‐polymer nanofibers with 6 and 12 wt% of ZIF‐8 crystallites embedded in the PVP polymer nanofiber, respectively. An et al.^68^ reported a variation of the slurry fabrication process for freestanding ZIF‐7/PAN nanofibers. In this process, the ZIF‐7 nanoparticles were crystallized in a PAN polymer solution from the metal precursor and the ligand before electrospinning (Figure 2d) instead of starting from a dispersion of crystalline MOF powder. By starting from the MOF precursors, the size of the ZIF‐7 particles could be controlled in the nanoscale well below the diameter of ZIF‐7/PAN nanofiber (Figure 2e,f) and the resulting MOF‐polymer composite reveals good mechanical properties and a flexible mat structure.

Zhang et al.^69^ demonstrated that a variety of MOFs with different functionalities could be combined with suitable polymers and processed as highly loaded dispersions by electrospinning, resulting in flexible MOF‐polymer composite nanofiber structures for use in air pollutant filters. Different types of MOFs with their specific functionalities are visualized in Figure 2g, including the ZIF‐8, UiO‐66‐NH_2_, MOF‐199, Mg‐MOF‐74 and their combination with the polymers PAN and PS. Nanofiber diameter, porosity, and pore size of the MOF‐polymer nanofibers could be controlled by optimizing the electrospinning conditions, such as applied electric voltage and flow rate, resulting in MOF nanofibers with different microstructures, as shown in Figure 2h. The ability to tailor the microstructure of nanofibers and the functionalization of the surface properties through MOFs are key variables for performance enhancements in the capturing of different types of pollutants in air filters.

**Figure 3:**
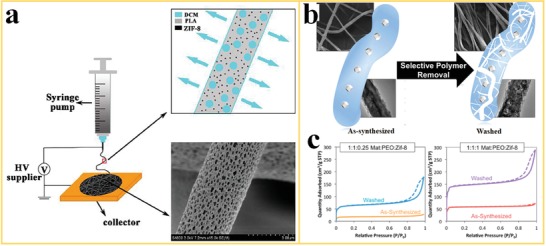
Electrospinning and post‐treatment approaches to create porosity in MOF‐polymer nanofibers: a) Fast solvent evaporation during electrospinning of PLA/ZIF‐8 nanofibers. Reproduced with permission.^70^ Copyright 2018, Elsevier. b) Selective removal of PEO polymer from ZIF‐8/matrimid‐PEO nanofibers by using solvent extraction. c) N_2_ isotherms for different ratios of Mat:PEO:ZIF‐8 in the “as‐synthesized” nanofibers and after PEO removal (“washed”). Reproduced with permission.^71^ Copyright 2017, American Chemical Society.

In MOF‐polymer composite nanofibers, the polymer phase acts as a binder supplying the structure with flexibility. However, the polymer might cover the surface and micropores of the MOF could negatively affect the performance in some specific applications, for example, the adsorption of gases on the MOFs. To overcome this problem, Dai et al.^70^ reported a special approach for electrospinning of porous PLA/ZIF‐8 nanofibers (poly(lactic acid), PLA) by utilizing fast solvent evaporation (**Figure** **3**a). In this work, CH_2_Cl_2_ was selected as solvent to disperse the ZIF‐8 into the PLA solution. The high volatility of the CH_2_Cl_2_ ensures fast evaporation during the flight of the electrospinning jet and the addition of ZIF‐8, which results in the formation of a large number of mesopores with large pore area fraction in the resulting PLA/ZIF‐8 nanofibers. The diameter and porous structure of the PLA/ZIF‐8 nanofibers could be efficiently adjusted by varying the loading fraction of the MOF particles.

Armstrong et al.^71^ presented an interesting approach for the fabrication of porous MOF nanofibers without affecting the distribution of the MOF particles in the fibrous structure (Figure 3b). A ZIF‐8 is first mixed with two polymers, polyimide Matrimid 5218 (Mat) and poly(ethylene oxide) (PEO), and the mixture is then used for electrospinning. Subsequently, the PEO polymer with a mass fraction of 50% is selectively removed from the nanofibers by washing with methanol. The N_2_ isotherms for as‐synthesized Mat:PEO:ZIF‐8 nanofibers with different ratio before (“unwashed”) and after PEO removal (“washed”) are shown in Figure 3c. The unwashed Mat:PEO:ZIF‐8 nanofibers display a Type I adsorption isotherm. After removal of PEO, a remarkable increase in N_2_ adsorption could be achieved by creation of pore channels in the polymer by washing (removal of PEO) and by the increase of the ZIF‐8 to polymer ratio. This process is similar to solvent extraction in injection molded ceramic parts before the thermal debinding step. Solvent extraction is a generic method which can be used to create porosity in a variety of MOF/polymer nanofiber composites with implications for the use in the field of filtration, adsorption, sensing, or catalysis.

### 2.2 Surface Decoration of Polymer Nanofibers with MOFs

The second route to produce hierarchical porous MOF‐polymer composite nanofibers is called “surface decoration” and describes the growth of MOF nanoparticles on the surface of nanofibers that have been produced by electrospinning. This route can overcome some of the drawbacks of the direct electrospinning route discussed in Section 2.1, such as poor dispersion of MOF particles in the polymers, blockage of MOF pores, or reduced mechanical properties, such as high brittleness due to MOF loading. In contrast to direct electrospinning, surface decoration allows the deposition of MOF layers with small crystallite size and controlled thickness on the nanofiber surface. For this purpose, a variety of processing methods or their combination have been applied, for example, layer‐by‐layer (LbL) assembly, sol‐gel processes, physical deposition methods, microwave heating, etc. The exposure of the MOF on the nanofiber surface is a great advantage for many applications, in which well‐accessible MOF particles are playing the role of “active sites.” The following methods can be distinguished to fabricate hierarchical porous MOF‐polymer structures via surface decoration: i) in situ growth and ii) phase transformation.

#### 2.2.1 In Situ Growth of MOFs on Polymer Nanofibers

During in situ growth, MOF precursors (usually a metal salt and an organic ligand) are dissolved in a suitable solvent and subsequently MOF nanoparticles are crystallized on the surface of a polymer nanofiber to obtain a MOF‐polymer nanofiber composite.^72^, ^73^ Typically, the nucleation, growth, and intergrowth of MOF crystals on the nanofibers occur at the same time. In situ growth of MOF on nanofibers can result in two types of MOF nanofiber architectures: freestanding or supported layers. The advantage of freestanding MOF‐polymer nanofiber layers is their higher flexibility and extended surface area compared to MOF layers that are supported by rigid substrates, such as metal plates, foams, or porous tubes.^74^, ^75^, ^76^, ^77^, ^78^, ^79^ The process of in situ growth of MOF structures on the surface of nanofibers can be categorized by the following approaches:

i) In “seed‐assisted growth” (also called “secondary growth”), MOF crystallite seeds are incorporated in a polymer nanofiber during electrospinning and the MOF growth is started by immersion of the seeded nanofibers in a MOF precursor solution.
ii) In another approach, one of the MOF precursors (the metal ion or the organic linker) is added to the polymer nanofiber via the electrospinning process and the MOF crystallization is initialized by immersion of the precursor‐polymer nanofiber in a solution of the other precursor.
iii) In direct solvo or hydrothermal growth, a MOF is directly grown on the surface of a polymer nanofiber by immersion in a MOF precursor solution (metal ion and organic ligand in a solvent). The polymer nanofiber usually needs to be activated for in situ growth by a nanofiber surface pretreatment to achieve sufficient attachment. The application of the MOF precursors can be done in one step (metal ion and organic ligand) or sequentially.

**Figure 4:**
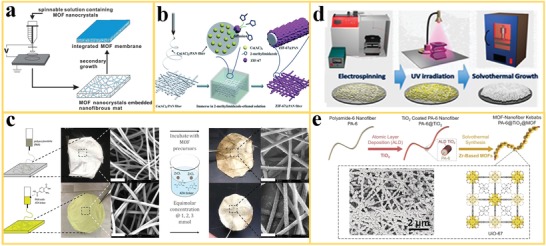
In situ growth approaches for the fabrication of MOF nanofibers. a) The growth of MOF crystals on polymer nanofibers with embedded MOF seeds. Reproduced with permission.^55^ Copyright 2012, Royal Society of Chemistry. b) Incorporation of metal ions (as Co(Ac)_2_) into PAN polymer nanofibers during electrospinning to enable the growth of ZIF‐67 on the nanofibers. Reproduced with permission.^81^ Copyright 2018, Royal Society of Chemistry. c) Addition of an organic linker (ATA) into PAN nanofibers during electrospinning for growth of UiO‐66‐NH_2_‐PAN nanofibers with improved MOF adherence and loading (scale bar is 3 µm). Reproduced with permission.^83^ Copyright 2017, American Chemical Society. d) Cross‐linking of PVCi via UV irradiation for stabilization of polymer nanofibers to enable MOF growth under solvothermal conditions. Reproduced with permission.^51^ Copyright 2015, American Chemical Society. e) Core–shell structure with an ultrathin ALD coating of TiO_2_ on PA‐6 nanofibers to enable the growth of Zr‐MOF with SEM images of resulting PA‐6@TiO_2_@UiO‐66‐NH_2_ nanofibers. Reproduced with permission.^92^ Copyright 2016, Wiley‐VCH.

All in situ growth approaches include multistep processing. Selected examples are discussed in the following section, including the use of specific fabrication techniques. In seed‐assisted growth, MOF seeds are embedded in the polymer nanofiber matrix to initiate further growth of the MOF particles. Wu et al.^55^ chose HKUST‐1, ZIF‐8, MIL‐101(Fe), and Zn_2_(bpdc)_2_(bpee) to demonstrate the effectiveness of MOF seeding in nanofibers to initiate subsequent intimate growth of MOF particles inside and on the surface of a MOF‐seeded polymer nanofiber structure by secondary growth to achieve a continuous, uniform, and defect‐free MOF membrane. The process is illustrated in **Figure** **4**a. The results illustrate that the electrospinning of composite nanofibers combined with secondary growth is a good approach to increase the loading fraction of MOF in MOF‐polymer nanofibers and to obtain continuous, freestanding MOF layers on porous nanofiber support structures. Similarly, Xu et al.^80^ developed Zn‐MOF/copolymer [P(St‐co‐TMSPMA)] (denoted as PST) nanofiber via electrospinning and anchoring of Zn‐MOF seed crystals on porous nanofiber supports to form Zn‐MOF/PST thin films.

Another approach is the electrospinning of a metal cation‐polymer mixture, usually as a metal salt mixed with a chelating polymer in a suitable solvent, and subsequent in situ growth of the desired MOF by immersing the obtained nanofiber in the solution of the suitable organic linker. Bian et al.^81^ used electrospinning to obtain a Co(AC)_2_/PAN nanofiber. The nanofiber was subsequently immersed in a solution of the organic linker 2‐methylimidazole (HmIM) in ethanol to grow a homogeneous layer of ZIF‐67 nanocrystals on the PAN nanofiber surfaces (Figure 4b). In a similar way, Talmoudi et al.^82^ used PVA as chelating polymer, Zn^2+^ as metal cation, and terephthalic acid as a linker to produce a MOF‐5‐PVA nanofiber with core–shell structures. Alternatively, Lu et al.^83^ reported the incorporation of the organic linker amino‐terephthalic acid (ATA) in the PAN nanofiber (in the electrospinning step) to promote the in situ growth of the MOF UiO‐66‐NH_2_ (UiO stands for Universitetet i Oslo) on PAN nanofibers for toxic chemical removal. This approach resulted in higher MOF loading and better adherence due to more homogeneous in situ growth of the MOF particles from the precursor solution on the PAN/ATA nanofiber surface compared to the growth on bare PAN nanofibers (Figure 4c).

MOFs can be synthesized directly on the surface of nanofibers (according to approach iii), but in most cases, the interface bonding between the MOF and the polymer is a challenge due to the different chemical properties. The activation of polymer nanofiber surfaces is a simple approach to anchor MOF particles to nanofiber surfaces during in situ growth from a MOF precursor solution. Laurila et al.^84^ proposed the surface modification of natural polymer‐based cellulose nanofibers by oxidizing the surface to generate anionic carboxyl groups or by adding carboxy methyl cellulose (CMC). Thereby, anchor sites for the metal ion were created which initiated subsequent MOF in situ growth on the polymer nanofiber surface. As a result, HKUST‐1 layers with good adhesion could be grown on pretreated cellulose nanofibers by immersion in the organic linker and the thickness was controlled by using a LBL assembly method. Similarly, Shangguan et al.^85^ used cross‐linked poly(acrylic acid)/PVA (PAA/PVA) nanofibers to decorate HKUST‐1 by a LBL assembly method. PAA/PVA nanofiber was selected as substrate because the hydroxyl and carboxyl groups of the polymers supply excellent binding sites for the Cu metal ions that are the centers for the further growth of the HKUST‐1. The PAA/PVA nanofiber surface is coated stepwise with the Cu(OAc)_2_ metal salt solution, followed by the organic ligand (trimesic acid, H_3_BTC) to prepare the HKUST‐1. LBL assembly is a feasible technique to produce nanolayers with controlled thickness on the surface of nanofibers but the multistep operation is time‐consuming.

Often, in situ growth of MOFs on polymer nanofibers is restricted by the stability of the polymeric nanofiber in the solvent which is required for the growth of the specific MOFs. Some MOFs are, for example, solvothermally prepared using DMF as solvent, which would dissolve polymer nanofibers made of PVP, PAN, or PVA. In addition, the crystallization of MOFs often requires relatively high reaction temperatures. Therefore, different approaches have been reported to stabilize polymer nanofiber structures for growth of MOFs. A simple approach (if applicable) is the replacement of a common solvent by a more appropriate solvent that is compatible with the polymeric nanofiber backbone and still allows the crystallization of the desired MOF structure. For example, Lu et al.^83^ conducted solvothermal growth of UiO‐66‐NH_2_ on PAN nanofibers in acetone (instead of DMF) because PAN is stable in acetone. Ji et al.^86^ reported in situ growth of Co‐MOFs on PAN nanofibers by using water as solvent.

For more severe conditions during in situ growth, cross‐linking of the polymers can be applied to stabilize the structure of polymers. This is a common approach which has been commonly used in polymer chemistry.^87^, ^88^, ^89^ Based on cross‐linking, Armstrong et al.^51^ reported the electrospinning of polymer poly(vinyl cinnamate) (PVCi) with seeds of UiO‐66 and a subsequent UV photocuring treatment for in situ growth of UiO‐66 (Figure 4d). The cross‐linking resulted in an improved thermal stability of the polymer nanofiber in DMF at high temperatures (to 120 °C) and allowed the decoration of the MOF on the surface of a stable PVCi polymer even under harsh solvothermal growth condition. Composite nanofibers consisting of inorganic/polymer hybrid materials (e.g., SiO_2_, ZnO, TiO_2_) have been developed to obtain more stable nanofiber substrate structures. Liu et al.^90^ demonstrated excellent stability of electrospun organic/inorganic PVA/PAA/SiO_2_ nanofibers under harsh solvothermal reaction condition in different solvents and at temperatures as high as 200 °C. Furthermore, these nanofiber structures revealed desirable mechanical properties such as malleability with a high breaking elongation. Liu et al.^91^ reported the excellent affinity of the surface of these PVA/PAA/SiO_2_ hybrid nanofiber for the in situ growth of different types of MOFs (HKUST‐1, MIL‐53(Al), ZIF‐8, and MIL‐88B(Fe)) due to a large number of available active surface hydroxyl and carboxyl groups for anchoring the MOF precursors.

Zhao et al.^92^ reported the coating of polymer nanofibers with metal oxides via atomic layer deposition (ALD) method to enable the growth of MOF structures. An ultrathin TiO_2_ layer was deposited on freestanding polyamide‐6 nanofibers to enhance the subsequent heterogeneous nucleation of Zr‐MOFs on the TiO_2_ surface (Figure 4e). The metal oxide serves as an effective protective coating around the nanofiber to avoid the dissolution and destruction of the polymer nanofiber structure during solvothermal synthesis. The TiO_2_ also provides the anchoring sites for the Zr‐MOF nucleation and growth. As a result, a continuous coverage of the nanofiber with Zr‐MOF particles was observed after in situ growth of different types of Zr‐MOFs, including UiO‐66, UiO‐66‐NH_2_, and UiO‐67. The scanning electron microscopy (SEM) image of PA‐6@TiO_2_@UiO‐66‐NH_2_ shows Zr‐MOF nanoparticles that are fully covering the nanofibers, indicating strong attachment to the substrates (Figure 4e). In contrast, when the same synthesis conditions are used for the direct growth of the UiO‐66‐NH_2_ directly on the parent PA‐6 nanofibers, surface incompatibility was observed.

#### 2.2.2 Phase Transformation to Decorate Polymer Nanofibers with MOF

**Figure 5:**
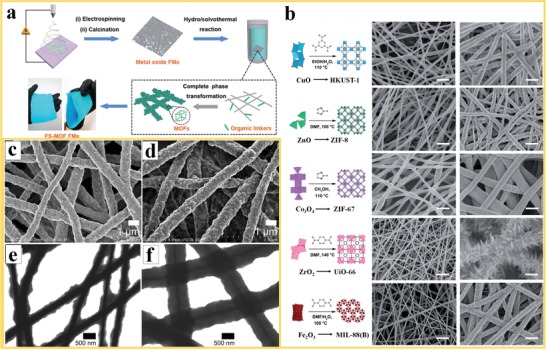
Phase conversion in the preparation of MOF nanofibers: a) Processing steps for the solvothermal conversion of metal oxide nanofibers into flexible self‐supported MOF nanofiber mats and b) different types of metal oxides that have been converted into MOF nanofibers by phase conversion and the corresponding SEM images of the microstructures (scale bar is 2 µm). Reproduced with permission.^93^ Copyright 2018, Royal Society of Chemistry. c,d) The SEM images of ZnO nanofibers and the corresponding ZIF‐8 nanofibers after gas phase transformation. e,f) TEM images of the ZnO/ZIF‐8 nanofibers after treatment at 150 and 200 °C. Reproduced with permission.^95^ Copyright 2018, Elsevier.

Phase transformation is another approach to grow MOFs on the surface of nanofiber structures, starting from a precursor (usually a metal oxide or hydroxide) which is incorporated in the surface of the polymer nanofiber. The precursor is converted into the MOF structure during in situ growth in a second step. A kinetic matching between the structure of the precursor and the targeted MOF structure is enabling the phase transformation. Liang et al.^93^ presented an approach to construct a variety of flexible self‐supported MOF nanofiber mats by phase conversion of metal oxide nanofibers through an optimized hydro/solvothermal synthesis. As illustrated in **Figure** **5**a, the metal oxide nanofiber is fabricated by electrospinning method into nanofibers followed by a calcination treatment. In the following step, the metal oxides on the nanofibers are converted into the respective MOF nanofiber structures by phase transformation under hydro‐ or solvothermal conditions in the presence of the organic linkers. The MOF crystallites are grown homogeneously on the surface of the nanofibers. The surface morphology varies depending on the type of reaction and the resulting MOF type. For example, for the conversion of ZrO_2_ into Zr‐MOFs, a large number of nanosheet arrays has been grown on the surface of the nanofibers (Figure 5b). Compared to the pristine ZrO_2_ nanofibers, a remarkable increase in diameter was observed after MOF growth, which can be explained by the volume expansion resulting from the conversion of the dense oxide phase to the less dense MOF by the addition of organic ligands. The phase conversion has been successfully demonstrated for the transformation of CuO, ZnO, Co_3_O_4_, ZrO_2_ and Fe_2_O_3_ to HKUST‐1, ZIF‐8, ZIF‐67, UiO‐66, and MIL‐88B(Fe), respectively (Figure 5b). Interestingly, these MOF nanofiber structures exhibit extra‐large surface area and remarkable high, open porosity that exceeded the reported values of MOF powders. Similarly, Bechelany et al.^94^ used ALD method for decoration of ZnO and Al_2_O_3_ on PAN nanofibers and subsequent solvothermal conversion under either conventional or microwave‐assisted heating to achieve phase transformation into thin ZIF‐8 and MIL‐53‐NH_2_ coatings on PAN nanofibers, respectively. With the combination of the deposition of the oxide by ALD, followed by microwave‐assisted heating and phase conversion, the fabrication process could be better controlled and resulted in a small MOF crystallite sizes and a very homogeneous coverage of the nanofibers.

Holopainen et al.^95^ reported the electrospinning of ZnO and aluminum‐doped ZnO with PVP and the subsequent gas‐phase conversion of the calcined nanofibers into ZnO/ZIF‐8 core/shell and pure ZIF‐8 nanofibers. The phase conversion was achieved by a solvent‐free thermal treatment in an autoclave under HmIM vapor at 150 and 200 °C, which preserved the fibrous structure. The SEM images (Figure 5c,d) reveal the nanofiber structure before and after phase conversion into ZIF‐8 with a size of about 300 nm on the nanofiber surface. In addition, the reaction proceeded from the nanofiber surfaces toward the core and the conversion rate for the forming of the ZIF‐8 increased with the reaction temperature. The resulting microstructures of the ZnO/ZIF‐8 core/shell nanofibers from gas‐phase conversion treatment at 150 and 200 °C for 18 h under HmIM atmosphere are shown in Figure 5e,f, respectively. By modulating the conversion parameters, ZIF‐8 nanofibers with very high Brunauer–Emmett–Teller (BET) specific surface area of1340 m^2^ g^−1^ could be achieved by this process.

### 2.3 Preparation of the Derivatives of MOF Nanofibers

Limited electrical conductivity and stability of MOFs have restricted their use in many energy and storage applications. MOFs as templates have been post‐treated to fabricate different kind of derivatives with high surface area, ordered pore structures, and uniform pore sizes for the use in energy and storage applications. Nevertheless, conventionally fabricated MOF derivatives still suffer from insufficient electrochemical performance related to a lack of electrical connectivity between the particles and limited mass and electron transfer. Therefore, considerable research efforts have been recently devoted to the development of the derivatives of MOF nanofibers to overcome these limitations. A few, selected examples of such type of nanostructures are discussed in this section and in Section 3.4 to highlight the opportunities to design these materials with advanced performances in energy and storage applications. A complete and detailed coverage of all the preparation routes, properties, and potential of MOF‐nanofiber derivatives in energy applications is exceeding the scope of this review. For further information, we refer the reader to a recent review dedicated to this topic by the group of Yamauchi.^60^

**Figure 6:**
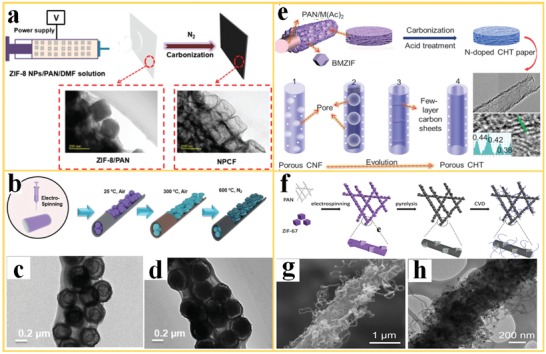
The processing and microstructure of derivatives of MOF nanofibers. a) Electrospinning and carbonization of ZIF‐8/PAN nanofibers into nanoporous carbon nanofibers (NPCF) and the respective TEM images. Reproduced with permission.^96^ Copyright 2018, Royal Society of Chemistry. b) The fabrication step to fabricate ysMnO*_x_*@NC, and the TEM images of c) preoxidized Mn‐BTC@PAN and d) ysMnO*_x_*@NC annealed at 600 °C. Reproduced with permission.^97^ Copyright 2019, Wiley‐VCH. e) CHTs with a large fraction of surface graphitization, including TEM image for the visualization of the enlarged *d*‐spacing. Reproduced with permission.^98^ Copyright 2017, Cell Press. f) Hierarchical porous carbon nanofibers and its g) SEM and h) TEM images showing the details of the microstructure with the CNF skeleton, including porous hollow carbon cube fillers, and CNTs attached to the surface. Reproduced with permission.^99^ Copyright 2018, Royal Society of Chemistry.

Wang et al.^96^ proposed the fabrication of nanoporous carbon nanofibers (NPFCs) by the heat treatment of ZIF‐8/PAN nanofibers in N_2_ atmosphere for the use in supercapacitors (**Figure** **6**a). Interestingly, major changes in the MOF structure, such as complete collapsing of the micropores, were not observed during the pyrolysis treatment but the parent ZIF‐8 particles transformed into hollow cubic carbon structures, entangled in a meso/microporous carbon nanofiber. This indicates that the PAN fiber stabilize the ZIF‐8 structure during carbonization, which is described as a confinement effect. The combination of a meso/microporous hierarchical structure of NPCs and high conductivity of the carbon nanofibers resulted in a very high value for gravimetric and volumetric capacitance of the NPCF electrode. Yang et al.^97^ reported the fabrication of yolk–shell MnO*_x_* nanoparticles confined in carbonized nanofibers (ysMnO*_x_*@NC) for use as anode materials in lithium‐ion batteries (LIB) (Figure 6b). The electrospinning method was applied first to structure the MOF Mn‐BTC (Mn‐1,3,5‐trimesic acid) into a polymer nanofiber matrix with a “beads‐in‐string structure.” The MOF‐polymer composite nanofibers were then preoxidized in air at 300 °C, leading to Mn‐BTC nanospheres with a multishell structure, displaying distinct spherical envelopes with a “ring‐in‐ring” morphology (Figure 6c). After further pyrolysis in N_2_ at 600 °C, a “yolk–shell” structure containing MnO*_x_* was obtained (Figure 6d). The MnO*_x_* structures confined in N‐doped carbon nanofibers offer double buffering for the volume fluctuations during prolonged charge/discharge cycling and thus maintain the structural stability and long‐term stability of the electrodes.

Chen et al.^98^ reported the fabrication of porous carbon nanofibers with N‐doping, high graphitization, and enlarged interlayer spacing to allow the intercalation of the relative large Na^+^‐ions in electrodes for high‐performance Na‐ion batteries, using MOF‐polymer nanofibers as a template (Figure 6e). For this purpose, a layer of a bimetallic MOF was grown on PAN nanofibers, containing metal acetates Zn(Ac)_2_ and Co(Ac)_2_. Upon pyrolysis of the nanofiber under high temperature and subsequent removal of residual metals by acid (HCl), N‐doped carbon hollow tubules (CHTs) were obtained, covered with a large fraction of MOF‐derived graphitic carbon with large interlayer spacing (0.38–0.44 nm). The resulting N‐doped porous CHT paper yielded in an outstanding cycling life (10 000 cycles), with high‐rate capabilities of Na^+^ intercalation and deintercalation and no decline in capacity. Li et al.^99^ developed a porous conductive nanofiber by MOF‐nanofiber carbonization and post‐treatment for similar application in Li‐S batteries. The process is schematically shown in Figure 6f. The ZIF‐67 nanoparticles are first dispersed in a PAN polymer solution and are afterward electrospun into composite nanofibers. The obtained ZIF‐67/PAN nanofibers are carbonized subsequently, resulting in ZIF‐67‐derived carbon cubes embedded in a carbon nanofiber. Finally, carbon nanotubes (CNTs) are grown on surface of the derived carbon fibers via a sequential chemical vapor deposition (CVD) process. The resulting hierarchical conductive fabric is composed of a carbon nanofiber skeleton, porous hollow carbon cube fillers, and CNTs, as shown in Figure 6g,h. This hybrid material exhibits high electrochemical activity with high conductivity, fast mass transfer, robust integrity, and strong sulfur confinement for use in Li‐S batteries, due to an exceptional hierarchical porous structure and the synergistic effect of the different building blocks of the composite.

## 3 Applications for MOF Nanofibers

### 3.1 Air Pollutant Filtration

**Figure 7:**
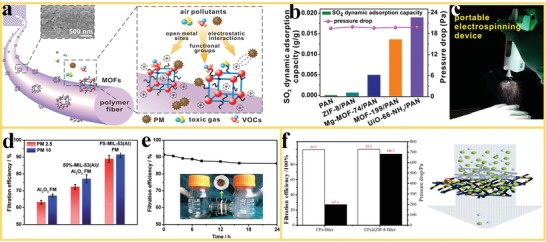
MOF nanofibers in air filtration. a) Proposed PM capture mechanism (inset: the SEM image of MOF nanofiber). b) SO_2_ dynamic adsorption on various type of filters. c) A photo of portable electrospinning device for deposition of MOF filters directly on a glove. Reproduced with permission.^69^ Copyright 2016, American Chemical Society. d) PM filtration efficiencies of MIL‐53(Al), 50%‐MIL‐53(Al)/Al_2_O_3_, and Al_2_O_3_ fibrous mats. e) Long‐term PM2.5 filtration efficiencies of the MIL‐53(Al) fibrous mats. Reproduced with permission.^93^ Copyright 2018, Royal Society of Chemistry. f) The air filtration performance of CFs@ZIF‐8 nanofiber filter (the white and black columns represent the filtration efficiency and the pressure drop, respectively). Reproduced with permission.^104^ Copyright 2018, Springer.

Air pollution, in the form of fine particulate matters (PMs) and toxic gases, has become an increasing public health hazard globally. To achieve efficient removal of PMs and toxic gases, the design of air filters with high selectivity and low cost has drawn considerable attention.^100^, ^101^, ^102^ Compared to conventional porous air filters, nanofiber filters structured by electrospinning offer higher performance due to large surface‐to‐volume ratio, tailored pore size, and high permeability. In addition, the functionalization of nanofiber surfaces with MOF nanoparticles offers an unique ability to enhance the removal of pollutants by combining air filtration and capture of poisonous pollutants in MOF‐nanofiber based filters. Large efforts have been dedicated to optimize the interaction between MOF and PMs or toxic gases to enhance air filtration efficiency and selectivities. For example, to remove fine PMs from the air, Zhang et al.^69^ developed various MOF‐PAN nanofiber filters with higher capture efficiencies and selectivities, as compared to pure PAN nanofibers (**Figure** **7**a). These performance improvements were attributed to the interactions between the PMs or toxic gases and the open metal sites or functional groups in the MOF nanoparticles. The capture efficiencies for the MOF/polymer filters achieved 88.33 ± 1.52% and 89.67 ± 1.33% for PM2.5 and PM10. Simultaneously, these fibrous filters enable the effective capture of SO_2_ with high selectivity. Compared with pure PAN filters, the UiO‐66‐NH_2_/PAN (0.019 g g^−1^) and MOF‐199/PAN (0.014 g g^−1^) nanofiber filters displayed a largely improved SO_2_ adsorption performance (Figure 7b). The results can be attributed to functional groups in the MOF structure, for example, amines, which are favorable for kinetic adsorption of acidic polar gas species. These type of filter materials can be coated on various substrates including lab coats, rubber gloves, and masks (Figure 7c).

The PMs capture efficiency often increases with the hydrophilicity and polarity of the filters. To improve the hydrophilicity, Wang et al.^103^ mixed acrylic acid (AA) with PLA and ZIF‐8 solution and electrospun the mixture into a nanofiber filter, followed by UV light exposure. The grafting of AA, triggered by UV light, effectively improved the hydrophilicity of the resulting AA‐PLA‐ZIF‐8 nanofiber filters due to the presence of the hydrophilic groups of —OH and —COOH in AA. Additionally, the ionization of —COOH produces negative charges and high polarity, which are beneficial for the capture of PM2.5. As a result, the PM2.5 capture efficiency of AA‐PLA‐ZIF‐8 filters was increased from 22% to 37%, as compared to the PLA‐ZIF‐8 nanofiber filters. Liang et al.^93^ reported MIL‐53(Al) nanofiber for PM removal. Remarkably, the FS‐MIL‐53(Al) nanofiber filters show excellent removal efficiencies of 91% and 89% for PM10 and PM2.5, respectively (Figure 7d). The reported performance is much higher than those of the 50%‐MIL‐53(Al)/Al_2_O_3_ mat (77% and 72%) and Al_2_O_3_ mat (67% and 63%). The long‐term filtration test shows a sufficient capture capability to block tobacco smoke during 24 h (Figure 7e). Similarly, Su et al.^104^ reported cellulose‐based air filter where ZIF‐8 nanoparticles were decorated on the surface of cellulose fibrous mats (CFs@ZIF‐8). The hybrid nanofibers were employed as “green” air filters for effective PM capture. Figure 7f shows an efficiency of 99.9% for the filtration with a CFs@ZIF‐8 nanofiber filter for simulated PM0.3, which meets the industrial N99 standard and was slightly above the filtration efficiency of a conventional CF. These results can be attributed to the presence of the ZIF‐8 nanocrystals in the CFs@ZIF‐8 nanofiber filter, which dramatically increased the specific surface area of the filter and the adsorption capacity for PMs as well as the interactions between the filter and PMs. A remarkable increase in the pressure drop of the CFs@ZIF‐8 nanofiber filter was also observed which can be explained by the ZIF‐8 nanoparticles clogging tiny openings in the filter structure. Thus, immobilized MOFs on nanofiber filters offer an improved performance in the capture of air pollutants, which is attributed to a combination of properties from the MOF and the nanofiber, such as large and highly open porosity, high specific surface area, polar–polar interaction with PMs, abundant cavities as well as efficient gas adsorption sites.

### 3.2 Water Treatment

**Figure 8:**
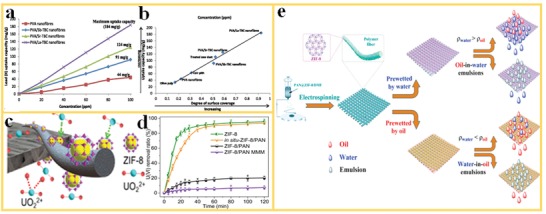
MOF‐polymer nanofibers for water treatment. a) Pb(II) uptake capacity of MOF‐PVA composite nanofibers. b) The comparison of maximum Pb(II) uptake capacities of different filter materials. Reproduced with permission.^109^ Copyright 2016, Springer. c) Adsorption of U(VI) ions on ZIF‐8 particles in ZIF‐8/PAN filters and d) the U(VI) removal rate for different filter materials. Reproduced with permission.^110^ Copyright 2018, American Chemical Society. e) Fabrication of PAN@ZIF‐8 membranes and the selective separation of oil/water mixtures and emulsions. Reproduced with permission.^116^ Copyright 2017, Elsevier.

Worldwide water pollution and scarcity of clean drinking water have led to a strong need for the development of energy‐savings and cost‐efficient water purification technologies.^105^, ^106^, ^107^ Thin‐film nanofiber membranes have been developed for various water treatment processes. These types of membranes offer high flux and large surface‐to‐volume ratio due to their hierarchical porous structure and interconnected pores. However, the majority of the applications of nanofiber membranes in water treatment is limited to the microfiltration range, due to the relatively large pore sizes (≈1 µm) of nanofiber structures. MOFs are particularly effective in the removal of contaminants with a much smaller size (e.g., heavy metals) by adsorption because of their high surface area and tailorable surface functionalities. For example, Efome et al.^108^ used MOF‐polymer nanofibers for removal of lead and mercury ions from aqueous solution. Water‐insoluble Fe(III)‐ and Zr(IV)‐MOF structures (MIL100‐Fe and MOF 808) were prepared and embedded in the PAN and polyvinylidene fluoride polymer to produce MOF‐polymer nanofiber membranes. The Fe‐MOF/PAN membranes showed high water flux of 348 L m^2^ h^−1^ with a permeance of 870 L m^2^ h^−1^ bar^−1^. The concentration of Pb (II) was below 10 ppb in the permeate, meeting the requirement for drinking water. Further investigations showed that the strong interactions between the Pb (II) and the Fe‐MOF contributed to the effective removal of the heavy metal, including competitive ion exchange (CIE), electrostatic interactions with the MOF crystals or polymer, and binding to open metal sites of the MOFs. In addition to the high removal efficiency, excellent durability of the MOF‐PAN nanofibers makes these membranes suitable for practical water treatment applications. For a similar application, Shooto et al.^109^ reported the fabrication of PVA/La‐TBC, PVA/Sr‐TBC, and PVA/La‐TBC nanofibers produced by the electrospinning method, where TBC is benzene 1,2,4,5‐tetracarboxylate. These hybrid nanofibers proved to be efficient adsorbents for heavy metals in contaminated water (**Figure** **8**a,b). The sorption data could be well fitted with Langmuir isotherms, indicating the homogeneous nature of the monolayer sorption of Pb(II) on the modified nanofibers. Wang et al.^110^ investigated ZIF‐8/PAN nanofibers for treating nuclear wastewater. The highly porous and open MOF‐polymer nanofiber structure with a large surface area exposes a large number of functionalized MOF sites for the adsorption of the metal ions from the wastewater. The ZIF‐8/PAN nanofibers exhibited a high adsorption capacity of U(VI) ions of 530.3 mg g^−1^ at a pH of 3.0. The removal of the U(VI) ions is due to the surface complexation of the 2‐Hmim linker in ZIF‐8 with U(VI), as indicated in Figure 8c. The adsorption kinetic measurements indicated that the removal rate of U(VI) for in situ grown ZIF‐8‐PAN nanofibers is comparable to that of pure ZIF‐8 (Figure 8d). In addition, the ZIF‐8/PAN composite nanofiber revealed a high selectivity of UO_2_^2+^‐ions over Ln^3+^‐ions.

Li et al.^111^ proposed the preparation of MOFs on electrospun silk nanofiber membranes to remove organic pollutants from wastewater. A biomineralization–biomimetic growth process was applied to form stable bio‐inorganic composites, leading to the full coverage of ZIF on the silk nanofibers. Relatively high mass loadings of 36 and 34 wt% were achieved for ZIF‐8 and ZIF‐67 by the use of the MOF‐polymer nanofibers. The studies demonstrate the advantages of MOF nanofibers for competitive ion exchange, utilizing MOF specific properties, such as electrostatic interactions and binding to open metal sites. Effective removal of congo red in wastewater treatment was also reported for ZIF‐8@PVA nanofibers by Fan et al.^112^ These examples demonstrate that MOF polymer nanofibers play an important role in water purification for the removal of heavy metal ions, organic dyes, or organic pollutants because of their unique nanofiber microstructure and tailorable surface properties.

Oil spill from different sources, for example, from tankers or oil drilling, is regarded as one of the most severe water pollution problems.^113^, ^114^, ^115^ Efficient ways of separation of oil from water are therefore required to protect the marine environment and is a large challenge. Cai et al.^116^ reported the development of novel materials and a separation membrane with unique wettability, composed of PAN‐ZIF‐8 nanofibers for the efficient separation of surfactant‐stabilized oil/water emulsions. The PAN‐ZIF‐8 nanofiber membrane can be tailored to superoleophobicity by prewetting with water and to superhydrophobicity after prewetting in oil (Figure 8e). In this way, both a high oil contact angle in water of 159^o^ and a high water contact angle in oil of 155° could be reached, which is favorable for improving fast separation dynamics. Accordingly, the hierarchically structured PAN‐ZIF‐8 nanofibers could effectively separate surfactant‐stabilized oil‐in‐water and water‐in‐oil emulsions with higher separation efficiency and flux compared to conventional membranes in pressure‐driven separation process.

Dai et al.^52^ reported ZIF‐8 in a PLA nanofiber matrix for oil/water separation. The PLA/ZIF‐8 nanofiber membranes showed increased oil wettability and significantly improved mechanical properties, as compared with pure PLA membranes. With 0.5 wt% ZIF‐8, the PLA/ZIF‐8 nanofiber membranes showed maximum tensile strength of 5.02 MPa and maximum strain of 88.81%. These results represent 81% improvement in tensile strength and 31% higher maximum strain, as compared to pure PLA nanofibers. The results thus indicate that fibrous MOF membranes are good candidates for highly efficient oil/water separation because of the fast oil wetting ability, large surface‐to‐volume ratio of the nanofibers, and the excellent adsorption effect of the porous MOF particles.

### 3.3 Gas Storage and Separation

The industrial separation of gas mixtures is of great importance for CO_2_ capture, natural gas sweetening, oxygen purification, hydrocarbon separation, or noble gas separation. Storage of gases, such as H_2_ or CH_4_, by adsorption on microporous materials in pressure vessels is developed as an alternative technology to replace fossil fuels in vehicles. Porous MOFs have been investigated for gas storage and separation because of extremely high accessible internal surface area, tunable surface chemistry, and pore sizes in the range of the kinetic diameter of different gas molecules.^117^, ^118^, ^119^ Interestingly, finely dispersed MOF particles have been structured into nanofibers to introduce effective adsorption sites. The resulting MOF nanofiber structures have potential to improve the performance of membranes or adsorption devices in gas storage and separation.

**Figure 9:**
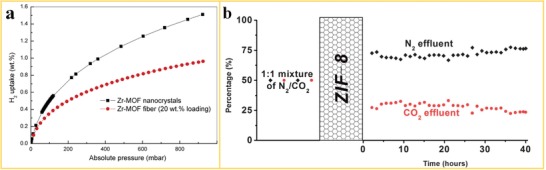
MOF‐polymer nanofibers in gas storage and separation. a) H_2_ sorption isotherms for pristine Zr‐MOF nanocrystals and Zr‐MOF nanofiber. Reproduced with permission.^120^ Copyright 2015, Elsevier. b) Separation performance of ZIF‐8 nanofiber for a 1:1 mixture of N_2_/CO_2_ as feed gas. Reproduced with permission.^55^ Copyright 2012, Royal Society of Chemistry.

Gao et al.^53^ developed flexible core–shell PAN@ZIF‐8 nanofibers for gas storage by secondary growth of MOF with a relatively high specific surface area of 983 m^2^ g^−1^ compared to 1219 m^2^ g^−1^ for the unstructured, pure ZIF‐8 powder. H_2_ and CO_2_ adsorption capacities of 8.1 and 13.3 m^3^ g^−1^ at 106 kPa have been measured for PAN@ZIF‐8 nanofibers. Ren et al.^120^ reported the preparation of MOF nanofibers for H_2_ storage with a Zr‐MOF (UiO‐66) and a Cr‐MOF (MIL‐101) embedded in a PAN polymer matrix by utilizing a vacuum degassing step to create the required open porosity in the PAN polymer matrix. Interestingly, structuring the MOF into MOF‐PAN nanofibers with a MOF mass fraction as low as 20 wt% could still achieve about 60% of the H_2_ uptake capacity of the pure MOF nanocrystals at 900 mbar (**Figure** **9**a). Zhao et al.^121^ measured the dynamic NH_3_ uptake of HKUST‐1 nanofibers. By decorating PP and PAN nanofibers with HKUST‐1, the surface areas of the MOF‐PP and MOF‐PAN nanofibers could be enlarged to 201 and 524 m^2^ g^−1^. Correspondingly, the MOF‐PP and MOF‐PAN nanofibers display 36 and 18 times higher dynamic loadings of NH_3_ compared to the uncoated PP and PAN nanofibers.

Wu et al.^55^ reported the growth of ZIF‐8 crystals into freestanding PVP nanofiber mats for CO_2_ separation from N_2_. The ZIF‐8‐PVP nanofiber structures with large open porosity revealed a very high permeability for both CO_2_ and N_2_. The membrane prepared by in situ growth showed higher CO_2_ adsorption capacity compared to a membrane prepared by direct electrospinning with MOF crystals embedded in the polymer nanofiber. The CO_2_ was enriched at the feed side and N_2_ was separated rapidly from the mixture because of the higher affinity of the ZIF‐8 to CO_2_ compared to N_2_ and a fast gas sorption‐diffusion‐separation process, which was enabled by the presence of a bimodal porosity in the ZIF‐8 fibrous membrane. As a result, a N_2_/CO_2_ separation factor of around 2.4 was achieved (Figure 9b). Fan et al.^65^ reported the use of ZIF‐8/PVP nanofibers as seeding structure to promote the in situ growth of a ZIF‐8 membrane on a macroporous SiO_2_ support for the separation of H_2_ from different gas mixtures. The separation factors of the binary gas mixtures of H_2_—CO_2_, H_2_—CH_4_, H_2_—N_2_ exceeded their corresponding Knudsen constant (≈3.74, 4.69, 2.83, respectively), indicating that a molecular sieving effect was achieved by using a ZIF‐8/PVP nanofiber membrane with intergrown ZIF‐8 crystals as a separation layer. Due to the large surface area, tailored surface chemistry, and well‐defined pore channels of MOFs, MOF nanofibers are very interesting materials for use in gas separation and storage.

### 3.4 Electrochemical Energy Storage and Conversion

**Figure 10:**
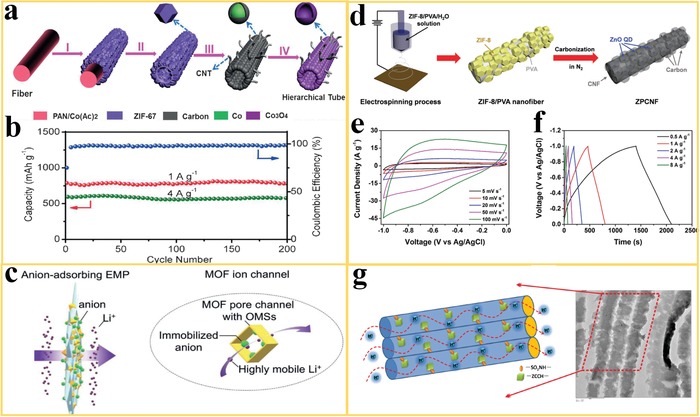
MOF nanofibers and derivatives used in electrochemical energy storage and conversion. a) Illustration of the fabrication steps of CNT/Co_3_O_4_ microtubes with I) the growth of ZIF‐67 onto the PAN‐Co(Ac)_2_ nanofiber and II) removal of the PAN‐Co(Ac)_2_ core. III) Heating treatment to convert ZIF‐67 tubular structures into CNT/Co‐carbon composite and IV) calcination to obtain the CNT/Co_3_O_4_ microtubes. b) Cycling performance and coulombic efficiency. Reproduced with permission.^124^ Copyright 2016, Wiley‐VCH. c) MOF‐PVA nanofiber used as separator for adsorbing anions and facilitating the transport of lithium ions. Reproduced with permission.^18^ Copyright 2019, Wiley‐VCH. d) Schematic illustration of the fabrication of ZPCNF and its e) CV curves and f) GCD curves. Reproduced with permission.^127^ Copyright 2017, Royal Society of Chemistry. g) A schematic of proton‐conduction in oriented MOF nanofibers and a TEM image of the structure. Reproduced with permission.^128^ Copyright 2014, Nature.

Advanced electrochemical devices for energy storage and conversions such as lithium batteries, supercapacitors, and fuel cells are promising technologies toward a sustainable energy future.^122^, ^123^, ^124^, ^125^ To overcome critical challenges for commercialization, the development of high‐performance electrode materials is a key toward fundamental advances in higher efficiency, better durability, and lower cost. Owing to the extremely high surface area and the flexibility in tuning pore size and functionality, the derivatives of MOF nanofiber (such as nanoporous carbon fibers, NPCFs) offer a combination of advantages to promote electrochemical activity. For example, Park et al.^126^ reported a novel carbonized MOF nanofiber electrode for lithium‐selenium (Li‐Se) batteries, which was fabricated by the carbonization of ZIF‐8/PAN nanofibers followed by subsequent chemical activation. The resulting electrode had a bimodal pore structure, micro‐ and mesopores in the carbon nanofibers (BP‐CNF), which plays an essential role in accommodating fine selenium particles to avoid the aggregation of these particles and facilitating mass transport. As a result, the BP‐CNF/Se electrodes achieved excellent coulombic efficiencies and discharge capacities, even at extremely high current densities. Chen et al.^124^ reported the preparation of hierarchical microtubular structures composed of Co_3_O_4_ hollow nanoparticles and carbon nanotubes for LIB, based on the heat treatment of ZIF‐67‐PAN nanofibers as template. The formation of the CNT/Co_3_O_4_ microtubes is visualized in **Figure** **10**a. The structure of the Co_3_O_4_ hollow nanoparticles and CNT subunits not only enables a short diffusion distance for fast diffusion of Li^+^ ions but also provides sufficient contact between the active material and the electrolyte for the rapid charge‐transfer. The ZIF‐67 derivative can alleviate the volume variation during the electrochemical reactions, and the CNTs can facilitate the fast electron transfer and avoid the aggregation of Co_3_O_4_ nanoparticles during cycling. As a result, the obtained CNT/Co_3_O_4_ microtubes show a high capacity of 782 and 577 mAh g^−1^ after 200 cycles at 1 and 4 A g^−1^, respectively (Figure 10b).

Zhang et al.^18^ reported novel composite membranes, consisting of MOF particles and PVA nanofibers as separator material for LIB. The separator in LIB acts as reservoir for electrolytes and plays a vital role in mediating the ions transfer toward better battery performance. The hydroxyl groups from PVA and dangling carboxylic acid groups from the UiO‐66 MOF form ester bonds via esterification, resulting in crosslinked MOF‐PVA networks. The MOF particles with open metal sites can spontaneously adsorb anions while allowing effective transport of lithium ions in the electrolyte (Figure 10c). This leads to significantly improved lithium‐ion transference number *t*_Li_^+^ (up to 0.79) and lithium‐ion conductivity. The incorporation of the MOF particles alleviates the decomposition of the electrolyte, enhances the electrode reaction kinetics, and reduces the interface resistance between the electrolyte and the electrodes. As a result, the MOF‐PVA nanofiber separator showed enhanced rate capability and cycling durability for use in conventional LIBs.

Lee et al.^127^ fabricated ZnO quantum dot‐decorated carbon nanofibers (ZnO QDs@carbon) by carbonization of electrospun ZIF‐8/PVA nanofibers for high‐performance electrodes in supercapacitors. During the carbonization, the PVA nanofibers served as precursor for the formation of a CNF structure and as an oxygen source to enable the transformation of the ZIF‐8 into finely dispersed ZnO QDs on the CNF surface (Figure 10d). These ZnO QDs‐decorated CNF (ZPCNF) based electrodes showed improved electrochemical performance compared with other ZnO‐based supercapacitors. The CNFs supply an efficient conductive pathway and the plum‐branch‐like ZnO QDs@carbons create an ion‐accessible area to enhance storage capacity and mass transfer through the electrode. As a result, the cyclic voltammetry (CV) and galvanostatic charge/discharge (GCD) measurements (Figure 10e,f) show an enhanced capacitance and rate capability for the ZPCNF electrodes compared to previously reported ZnO composite materials.

As the heart of a direct methane fuel cell, a proton exchange membrane plays an essential role in determining fuel cell durability, methanol crossover, and proton conductivity. Ideally, proton‐exchange membrane fuel cells (PEMFCs) should be operated at high temperature (>100 °C) to eliminate CO poisoning and to promote the electrochemical reactions. However, perfluorinated sulfonic acid (PFSA) membranes, such as Nafion, use H_2_O molecules as the vehicles to transport protons, which limits the fuel cell operation temperature to below 100 °C. Wu et al.^128^ proposed the design of electrospun Zn‐MOFs/SPPESK (sulfonated poly(phthalazinone ether sulfone ketone)) nanofiber membranes for PEMFCs. The Zn‐MOF with the formula Zn_2_(C_2_O_4_)(C_2_N_4_H_3_)_2_(H_2_O)_0.5_ (ZCCH) shows significant proton conductivity at high temperature and anhydrous conditions because protonic charge carriers (water, acids, heterocycles) can be incorporated in the pores. The proton‐conductive Zn‐MOF was mixed with the polymer SPPESK and electrospun into a highly oriented nanofiber membrane, to construct a continuous proton‐conducting path (Figure 10g). The Zn‐MOF/SPPESK nanofiber membrane resulted in a remarkably high proton conductivity of about 8.2 × 10^−2^ S cm^−1^ at 160 °C. Moreover, this composite membrane had a methanol permeability of 0.707 × 10^−7^ cm^2^ s^−1^, which is only about 6% of Nafion 115. This type of MOF‐polymer nanofiber membrane with high proton conduction can pave the way to the development of high‐temperature PEMFCs with enhanced CO tolerance.

**Figure 11:**
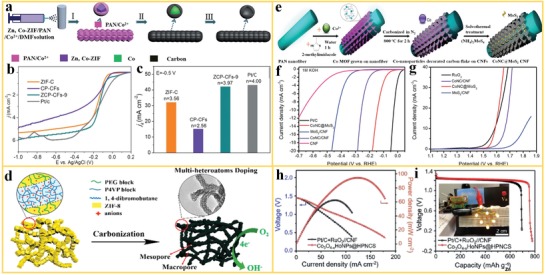
The derivatives of MOF nanofibers used in electrocatalysis. a) Schematic of the preparation and structure of ZCP‐CNF derived from ZnCo‐ZIF/PAN nanofiber for ORR. b,c) Polarization curves and kinetic‐limiting current density of various electrocatalysts. Reproduced with permission.^131^ Copyright 2017, Royal Society of Chemistry. d) Carbonization of a ZIF‐8/polymer nanofiber into a hierarchical porous, worm‐like CNF architecture with multi‐heteroatom doping for use in ORR. Reproduced with permission.^133^ Copyright 2017, American Chemical Society. e) The fabrication of a CoNC@MoS_2_/CNF hybrid material as bifunctional HER and OER electrocatalyst. f,g) Performance of the hybrid material with polarization curves.^86^ Copyright 2017, Royal Society of Chemistry. h) Discharging polarization curves and corresponding power plots and i) voltage–capacity curves of derivative of Co‐ZIF‐L/PAN nanofibers for a noble‐metal‐based Zn‐air battery. Adapted with permission.^73^ Copyright 2019, Wiley‐VCH.

Electrocatalysis, which occurs at the electrode–electrolyte interface, is an important process for the catalysis of electrochemical reactions in various energy conversion and storage technologies, such as fuel cells, metal‐air batteries, and electrolyzers for water splitting.^129^ Electrodes for hydrogen evolution reaction (HER), oxygen reduction reaction (ORR), and oxygen evolution reaction (OER) have been widely investigated recently. These processes involve multistep electron transfer steps and suffer from sluggish kinetics.^129^, ^130^, ^131^, ^132^, ^133^, ^134^ Some efforts have been devoted to the development of high‐performance noble metal‐free based electrocatalysts to reduce costs and to improve the durability of the electrodes. In this respect, the derivatives of MOF nanofibers are promising materials for electrodes in electrocatalysis. Liu et al.^131^ reported the synthesis and characterization of Zn, Co‐ZIF nanofiber derived carbon nanofiber (ZCP‐CFs) for ORR. The ZCP‐CFs were prepared by electrospinning, carbonization, and subsequent acid etching treatment from ZnCo‐ZIF/PAN nanofibers, as visualized in **Figure** **11**a. The carbon nanofibers with high degree of graphitization resulted in synergistic structural effects, such as large surface area, uniformly distributed active sites, and increased electrical conductivity. The combination of these effects contributed to an excellent ORR activity. Rotating‐disk electrode (RDE) measurements were used for investigating the ORR kinetics. The results revealed that a derivative of MOF nanofiber based electrode (ZCP‐CFs‐9) showed a lower overpotential of −0.135 V versus Ag/AgCl (Figure 11b) and a high current density of 42 mA cm^−2^ (Figure 11c) compared to other non‐noble metal electrocatalysts. Huang et al. reported the fabrication of multi‐heteroatom‐doped porous carbon structures for use in ORR derived from ZIF‐8 nanofibers. These special structures resulted in internal positively charged polymeric wormlike micelles and a microporous layered structure derived from the MOF nanofiber template.^133^ After pyrolysis, the obtained N, B, P, S‐doped porous carbon nanofiber network showed a large number of mesopores and macropores in the network (Figure 10d), which effectively exposed the active sites to the reaction and promoted fast mass transfer. The homogeneous multi‐heteroatom doping improved kinetics and resulted in excellent ORR performance, which is comparable to commercial Pt/C based electrodes.

Ji et al.^86^ proposed the fabrication of bifunctional OER and HER electrocatalysts by electrospinning of MOF/polymer precursors for use in water splitting. As shown in Figure 10e, a Co‐MOF was in situ grown on a PAN nanofiber and subsequently treated by pyrolysis, leading to the formation of Co‐doped carbon flakes grafted on carbon nanofiber (CoNC/CNF). Finally, MoS_2_ was successfully grown on the surface of the carbon flakes, leading to the formation of CoNC@MoS_2_/CNF. A unique architecture with interconnected vine‐like CNFs was observed for CoNC@MoS_2_/CNF, which contributed to improved electrocatalytic performance in HER (Figure 11f) and OER (Figure 11g) with low overpotentials. In addition, no obvious decrease in current density was observed after 200 000 s, indicating excellent stability in alkaline solution. Moreover, the combined use of the bifunctional CoNC@MoS_2_/CNF electrode both in the HER and the OER led to a high performance in water splitting that was comparable to a cell with Pt/C and RuO_2_ electrodes. Ji et al.^73^ reported the preparation of hollow Co_3_O_4_ particles with abundant oxygen vacancies that were embedded in a porous N‐doped carbon structure via pyrolysis of Co‐ZIF‐L/PAN nanofibers for use in zinc‐air batteries. Here, the structure and defect control is an effective way to optimize the activity and stability of the electrocatalyst. The number of oxygen vacancies in the embedded Co_3_O_4_ particles was regulated by the degree of Kirkendall oxidation, which can result in a large number of active sites, resulting in low reversible oxygen overpotential for ORR/OER. This type of hollow Co_3_O_4_ particle catalyst in a N‐doped hierarchical carbon structure on carbon nanofibers showed a high power density of 94.1 mW cm^−2^ and a capacity of 779.36 m Ah g_zn_^−1^ in a home‐made Zn‐air battery (Figure 11h,i). This is a promising type of electrocatalyst architecture to substitute conventional noble metal catalysts in the cathodic oxygen reaction. Structures based on the derivatives of MOF nanofibers offer synergistic effects that can largely contribute to improvements in the activity, efficiency, and stability of catalysts in electrocatalysis. The improvement can be attributed to a hierarchical porous MOF derived structure that effectively exposes the active sites and promotes fast mass transfer. In addition, homogeneous multi‐heteroatom doping via MOF‐derived structures can improve reaction kinetics and increase the catalytic activity. The integration of such structures into suitable carbon nanofiber structures improves conductivity and structural stability.

### 3.5 Heterogeneous Catalysis

**Figure 12:**
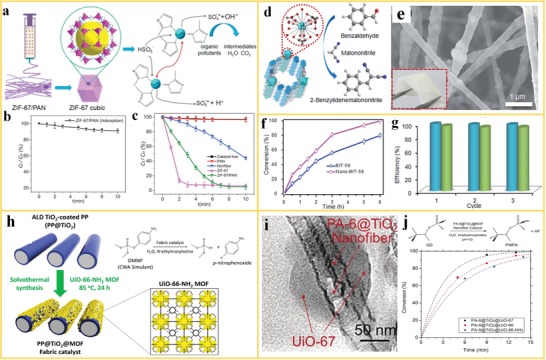
MOF nanofibers in heterogeneous catalysis. a) A schematic of electrospinning, structure, and reaction of ZIF‐67/PAN nanofibers in oxidative decomposition of organic pollutants, showing the activation of peroxymonosulfate and generation of sulfate radicals. b) Decolorization of AY using ZIF‐67/PAN via adsorption, c) PMS activation and yellow‐17 (AY) pollutant degradation on PAN, ZIF‐67, and ZIF‐67/PAN nanofibers. Reproduced with permission.^138^ Copyright 2017, Elsevier. d) The schematic representation of the use of BIT‐58 in the catalytic reaction of benzaldehyde to 2‐benzylidenemalononitrile and e) photo and SEM images of the nano‐BIT‐58/PAN nanofiber. f) Catalytic performance of BIT‐58 and nano‐BIT‐58. g) Catalytic performances of the film during three catalytic cycles. Reproduced with permission.^139^ Copyright 2018, Royal Society of Chemistry. h) PA‐6@TiO_2_@UiO‐66 nanofiber used for the degradation of highly toxic CWAs. Reproduced with permission.^140^ Copyright 2017, American Chemical Society. i) TEM images of a PA‐6@TiO_2_@UiO‐67 nanofiber. j) Conversion of nerve agent soman (GD) using Zr‐MOFs nanofiber versus reaction time. Reproduced with permission.^92^ Copyright 2016, Wiley‐VCH.

Heterogeneous catalysis, in which the catalyst occupies a different phase than the reactants and products, enables large‐scale production, and selective product formation. MOFs are regarded as single‐site catalysts because the coordinatively unsaturated metal ions, substituents at the organic linkers or guest species located inside the framework can play the role of the active site.^135^, ^136^, ^137^ Nevertheless, MOF crystals are fragile and difficult to use as stable catalysts in heterogeneous catalysis, which may result in loss of catalyst even under relatively mild reaction conditions. Electrospinning provides a promising way for immobilizing MOFs into polymer nanofiber scaffolds, to ensure a large number of exposed catalytic sites with excellent stability. Wang et al.^138^ reported the use of ZIF‐67/PAN nanofibers for activation of peroxymonosulfate (PMS) as a catalyst to degrade yellow‐17 (AY) color pigment (**Figure** **12**a). The effect of the catalyst dosage, reaction temperature, solution pH, and doping on the degradation of AY on the PMS was systemically investigated. As shown in Figure 12b,c, the ZIF‐67/PAN nanofibers can degrade 95.1% ZY (500 mg L^−1^) within 10 min. The catalytic sites are protected by the rigid framework of ZIF‐67 embedded in a flexible nanofiber structure. This architecture ensures a stable catalytic activity and the catalyst can easily be separated (recycled) after the reaction is completed. Furthermore, the work demonstrated that ZIF‐67/PAN nanofibers are an efficient catalyst for the oxidative catalytic degradation of other pollutants for the recovery of waste water, proving the decomposition of 68.3% of tetracycline (TC), 100% of bisphenol A (BPA), and 98.3% of rhodamine B (RhB) by ZIF‐67/PAN.

Chen et al.^139^ reported the preparation of a lanthanide‐based MOF nanofiber structure in the Knoevenagel condensation, utilizing the MOF BIT‐58 (BIT stands for Beijing Institute of Technology). This MOF has a catalytic active site with a Ce^3+^ metal center and 1,3,5‐tris(4‐carboxyphenyl)benzene (BTB) as the organic ligand (Figure 12d). The BIT‐58/PAN nanofibers displayed an interwoven network where the BIT‐58 catalyst is embedded in the polymer matrix (Figure 12e). The open and accessible porosity of this MOF nanofiber structures exposes the active Lewis acid sites on the BIT‐58 and the nanofiber matrix improves the structural stability. The BIT‐58/PAN nanofibers displayed a high catalytic efficiency in the Knoevenagel condensation reaction with a 100% conversion efficiency in 6 h (Figure 12f). Furthermore, the reusability of the MOF‐polymer nanofiber catalyst has been tested with no apparent loss in catalyst efficiency in a three‐cycle test (Figure 12g) demonstrating high durability of the catalyst structure.

Lee et al.^140^ reported a series of Zr‐MOF nanofibers for fast catalytic degradation of chemical warfare agents (CWAs) simulant. Different layers of inorganic materials, including Al_2_O_3_, ZnO, or TiO_2_, were coated on polypropylene (PP) nanofibers. Subsequently, UiO‐66‐NH_2_ was grown on the surface of the metal oxide coated nanofibers. The hydrolysis of a simulant material for CWAs, 4‐nitrophenyl phosphate (DMNP), was used as an example to evaluate the catalytic performance of MOFs nanofibers for this application (Figure 12h). As compared to Polyamide‐6 (PA‐6) and PA‐6@TiO_2_, the MOF‐nanofiber structures of PA‐6@TiO_2_@UiO‐66‐NH_2_ and PA‐6@TiO_2_@UiO‐67 both resulted in a short half‐life of DMNP (7.3 and 7.4 min, respectively) and high conversion rate (>90%) within 1 h. As compared with that of PP nanofiber, the TiO_2_ coating enabled a more effective and uniform MOF nanofiber adhesion, resulting in a rapid catalytic degradation rate. These results show that Zr‐MOFs have the capability of catalytically hydrolyze phosphonate ester bonds. In addition, Zhao et al.^92^ reported the use of PA‐6@TiO_2_@UiO‐67 nanofibers for the catalytic degradation of the nerve agent soman (O‐pinacolyl methylphosphonofluoridate, GD) (Figure 12i,j). The Zr‐MOF nanofibers showed a fast GD destruction (*t*_1/2_, 4 min) and high conversion (80%) within 10 min, due to the optimal microstructure of UiO‐67 that allowed easy diffusion of reactants into the pores and fast reaction at the catalytic active sites of the frameworks. This work demonstrated, for the first time, an effective approach to decompose a real CWA compound using MOF nanofibers.

**Figure 13:**
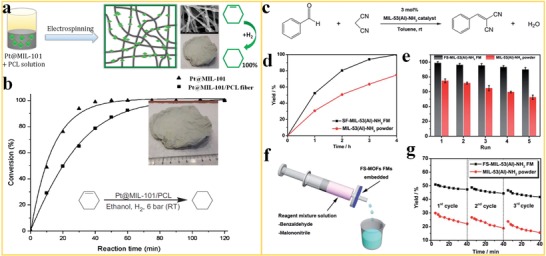
MOF nanofibers used in heterogeneous catalysis. a) Pt@MIL‐101 embedded in a PCL matrix as a “catalytic carpet” for highly efficient catalysis. b) The conversion of cyclohexene to cyclohexane using Pt@MIL‐101/PCL and Pt@MIL‐101 as a catalyst. Reproduced with permission.^22^ Copyright 2018, Elsevier. c) The Knoevenagel condensation of benzaldehyde with malononitrile catalyzed by MIL‐53(Al)‐NH_2_. d,e) Time–yield plots and yields at different runs of the catalysis by the MOF nanofiber and powder. f) Illustration of the plug‐flow reactor. g) Time‐dependent yield at different cycles catalyzed by the MOF nanofiber and powder in a plug‐flow reactor. Reproduced with permission.^93^ Copyright 2018, Royal Society of Chemistry.

In order to improve the stability of catalysts and to avoid leaching problems, Leus et al.^22^ reported the fabrication of a “catalytic carpet” by electrospinning a mixture of Pt@MIL‐101(Cr) and a poly‐є‐caprolactone (PCL) polymer for use in catalysis (**Figure** **13**a). The obtained nanofiber architecture revealed a high degree of dispersion of Pt@MIL‐101 immobilized in the PCL matrix. This “catalytic carpet” was highly efficient for hydrogenation of cyclohexene. In addition, the fibrous catalyst could be recovered just within seconds after catalysis, without any weight loss for the next catalytic runs. Full conversion can be obtained within 1.5 h, in which a substantial fraction (>65%) of the total amount of Pt‐atoms participating in the catalytic reaction (Figure 13b). Most importantly, the Pt@MIL‐101‐PCL catalyst can be recycled for at least four runs without detectable Cr and Pt leaching. Complete recovery was achieved with zero weight loss while fully preserving the nanofiber structure. The Knoevenagel condensation, as a typical weak base‐catalyzed model reaction for the synthesis of fine chemicals, attracted considerable interest. Liang et al.^93^ reported about MIL‐53(Al)‐NH_2_ nanofibers for the Knoevenagel condensation (Figure 13c). The Knoevenagel condensation catalyzed by the MIL‐53(Al)‐NH_2_ nanofiber structure proceeded very fast, with a yield of 100% within 4 h (Figure 13d). The MIL‐53(Al)‐NH_2_ nanofiber revealed excellent durability with only 10% loss of yield from 100% to about 90% after five cycles (Figure 13e) whereas an obvious degradation of activity was observed on the MIL‐53(Al)‐NH_2_ powder. In addition, a plug‐flow reactor was fabricated (Figure 13f), which started with a yield of around 50% and sustained a high yield for more than 45 min for three successive cycles (Figure 13g). The excellent performance of the MIL‐53(Al)‐NH_2_ nanofiber can be attributed to a substantial increase in surface area and highly exposed active MOF sites when MIL‐53(Al)‐NH_2_ was structured into nanofibers. As conclusion, in heterogeneous catalysis MOF nanofiber structures can significantly reduce the loss of catalytic sites (MOFs), improve durability, and enhance mass transfer due to efficient fixation of the catalytic sites, high surface area, and a hierarchical porous catalyst support structure.

## 4 Conclusions

Intensive research has been reported for the structuring of MOFs into hierarchical porous nanofibers. Two routes are developed: i) direct electrospinning of a MOF‐polymer dispersion or ii) surface decoration of MOF on the surface of polymer nanofibers. The developed hierarchical nanofiber architectures show interesting properties, which can be utilized for various energy and environmental applications. In many areas, including gas separation, air filtration, water treatment, and heterogeneous catalysis, the research will most likely focus on the development of MOF‐polymer nanofibers because the functionality of the original MOF structures is required for the application. In other application, mostly those related to electrochemical energy conversion, for example, electrodes in batteries or fuel cells, sufficiently high electronic conductivity is required. It is expected that the research in these areas would be mostly devoted to the derivatives of MOF nanofibers, such as MOF nanofiber derived 1D porous or hollow carbon‐based nanofibers.

Considering the complexity of structuring MOF nanofibrous architectures and the rapid progress in energy and environmental applications, a couple of challenges could be addressed in the future: i) Investigation of a range of polymers that can be processed into nanofiber systems and can withstand MOF synthesis conditions under harsh conditions (temperature, aggressive solvents) during MOF decoration. (ii) Exploring the implementation of postfabrication processes for MOFs nanofiber structures, which are already used for processing of conventional nonwoven fibers in textile industry or related industries. Examples are vacuum forming of fiber blankets, calendaring, or needle punching.^141^, ^142^, ^143^ (iii) MOF‐polymer composites are a relatively new class of architectures and therefore more theoretical investigations would be desirable that could explain the relationship between the structure and the performance for relevant applications.

Commercial products of MOF nanofibers have so far not been reported in energy and environmental applications. A combination of relatively high costs for the expensive raw materials (MOFs) and for complex fabrication processes (relative low production rates in electrospinning and MOF growth) could be factors for inhibiting the commercialization of MOF nanofiber products. MOF nanofiber products are expected to enter markets first in those areas in which they can provide significant performance improvements and create high value. Examples are the efficient capture/removal of emerging pollutants from air or water, replacement of expensive noble metals in electrocatalysis, high value biomedical and healthcare products, or drug or enzyme delivery.

In conclusion, electrospinning is a new path for structuring of functional MOFs into hierarchical nanofiber structures, which allows the creation of a range of fascinating new properties. We hope that this review can guide and inspire researchers to do further work on MOF nanofibers, which should eventually lead to functional MOF nanofibers used in energy and environmental applications.

## Conflict of Interest

The authors declare no conflict of interest.
